# Stellenwert des „Stone-heart“-Phänomens bei Herz-Kreislauf-Stillstand

**DOI:** 10.1007/s00113-020-00856-w

**Published:** 2020-08-15

**Authors:** J. Unseld, Patrick Pflüger, Maximilian Landeg, Michael Dommasch, K.‑G. Kanz, V. Bogner-Flatz

**Affiliations:** 1 Klinik für Allgemeine, Unfall- und Wiederherstellungschirurgie, Klinikum der LMU München, München, Deutschland; 2 Klinik und Poliklinik für Unfallchirurgie, Klinikum rechts der Isar, Technische Universität München, München, Deutschland; 3 Klinik und Poliklinik für Innere Medizin I, Klinikum rechts der Isar, Technische Universität München, München, Deutschland

**Keywords:** Reanimation, „Myocardial stunning“, Thorakotomie, Systolische Myokardkontraktur, Hypoxie, Hypoxie, Cardiopulmonary resuscitation, Myocardial stunning, Thoracotomy, Systolic myocardial contraction, Hypoxia

## Abstract

Der Begriff Stone heart ist definiert als systolische Kontraktur des Herzens und wird auch als kontraktiler Herzstillstand bezeichnet. Er wurde erstmals 1972 durch den US-amerikanischen Herzchirurgen Denton Cooley bei Patienten mit Bypass-Operation beschrieben. Das Stone heart ist meist Folge eines prolongierten Herz-Kreislauf-Stillstands, welcher zu einer Anoxie bzw. Hypoxie des Myokards führt. Es wird über 3 Traumapatienten berichtet, welche nach kardiopulmonaler Reanimation in der postmortalen Computertomographie (CT) ein Stone-heart-Phänomen zeigten.

## Abstract (en)

The stone heart syndrome is defined as an ischemic systolic contracture of the heart and also termed contractile cardiac arrest. It was first described in 1972 by the American cardiac surgeon Denton Cooley, who observed this phenomenon during bypass surgery. It is mostly the result of prolonged cardiac arrest where myocardial cells suffer hypoxia or anoxia. Insufficient forward blood flow and a decreased pressure gradient in the central aorta lead to reduced coronary perfusion. The resulting anaerobic metabolism causes an ischemic contracture as described in the stone heart syndrome. This article presents three cases of patients with traumatic cardiac arrest (TCA) and myocardial contracture in postmortem computed tomography (PMCT) and discuss the origins of the stone heart syndrome as well as its implications in cardiopulmonary resuscitation.

## Falldarstellung 1

### Anamnese

Ein 31-jähriger Patient kollidierte als Fahrer seines Pkw mit ca. 100 km/h frontal mit einem Baum und war im Fahrzeug eingeklemmt. Bei Eintreffen des Rettungsdienstes war er bewusstlos (GCS 3), apnoisch und asystolisch. Nach Rettung wurde er vor Ort durch den Rettungsdienst kardiopulmonal reanimiert, endotracheal intubiert und erhielt zudem beidseits eine Thoraxdrainage.

### Befund

Es erfolgte die Aufnahme in den Schockraum unter laufender kardiopulmonaler Reanimation durch die Notarztbesatzung. Die endotracheale Tubuslage war korrekt, es zeigte sich kein Spannungspneumothorax bei bereits liegenden Thoraxdrainagen beidseits. Im E‑FAST konnten keine Perikardtamponade oder intraabdominelle Blutung festgestellt werden. Der Patient hatte keine weiteren offensichtlichen Extremitätenverletzungen.

### Therapie und Verlauf

Entsprechend dem ATLS-Schema erfolgte das weitere Schockraummanagement. Der Patient zeigte kein Ansprechen auf die durchgeführte Therapie mit Volumengabe und Vasopressoren; das Wiedereinsetzen eines Spontankreislaufs (ROSC) blieb aus. In Zusammenschau der Befunde wurden durch das Team im Schockraum nach insgesamt 40 min Reanimation die Maßnahmen eingestellt.

### Postmortale Bildgebung

**Figure 1:**
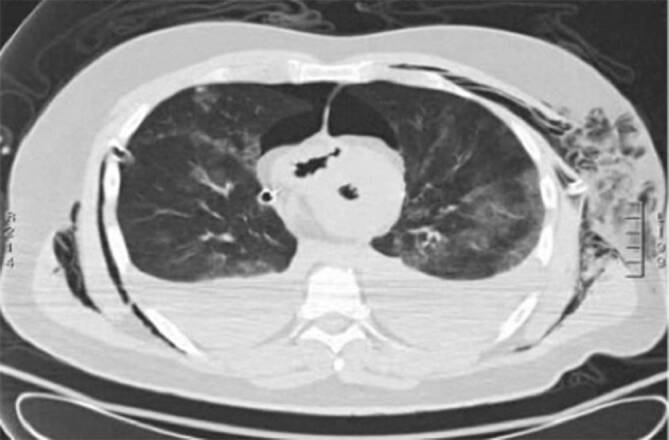

In der postmortalen Computertomographie (pmCT) zeigten sich ein Hirnödem mit Herniation und intrakraniellen Lufteinschlüssen sowie eine Mittelgesichtsfraktur LeFort-Typ III. Zudem bilaterale Hämothoraces mit Rippenserienfrakturen beidseits. Außerdem zeigte das CT eine Wandverdickung des linken Ventrikels bei gleichzeitig reduziertem linksventrikulärem Volumen im Sinne eines Stone-heart-Phänomens (Abb. 1).

## Falldarstellung 2

### Anamnese

Ein 31-jähriger Patient war über ein Treppengeländer ca. 10 m in die Tiefe gestürzt. Initial war der Patient kardiopulmonal stabil, wurde aber während des Transports in die Klinik im Rettungstransportwagen reanimationspflichtig. Es erfolgten die endotracheale Intubation und bei abgeschwächtem Atemgeräusch rechts die Anlage einer Thoraxdrainage rechtsseitig.

### Befund

Aufnahme in den Schockraum unter laufender kardiopulmonaler Reanimation durch die Notarztbesatzung. Die korrekte endotracheale Tubuslage wurde verifiziert. Es zeigte sich ein Spannungspneumothorax bei bereits liegender Thoraxdrainage rechts. Daraufhin erfolgte die Anlage einer zweiten Thoraxdrainage rechts. Im E‑FAST konnte keine Perikardtamponade oder intraabdominelle Blutung festgestellt werden. Der Patient hatte keine weiteren offensichtlichen Extremitätenverletzungen.

### Therapie und Verlauf

Entsprechend dem ATLS-Schema erfolgte das weitere Schockraummanagement. Die Ganzkörper-CT mit mechanischer Reanimationshilfe (LUCAS®, Chest Compression System, Physio-Control/Jolife AB, Schweden) zeigte oben genannten Befund. Der Patient zeigte kein Ansprechen auf die durchgeführte Therapie; ein ROSC blieb aus. In Zusammenschau der Befunde wurden durch das Team im Schockraum die Maßnahmen bei über einstündiger Reanimationszeit terminiert.

### Postmortale Bildgebung

**Figure 2:**
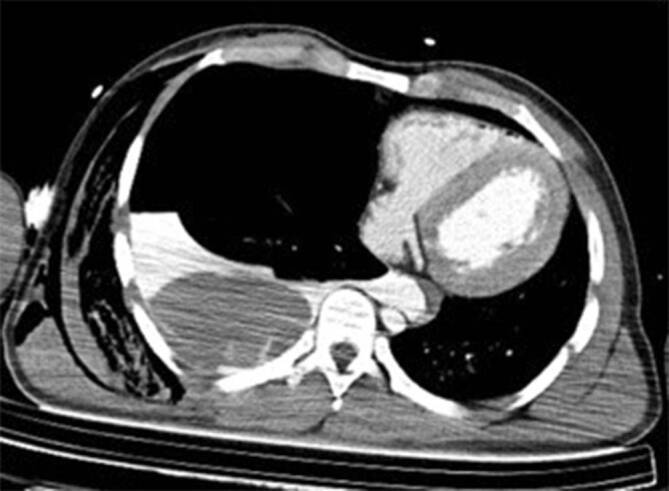

Im pmCT zeigte sich ausgedehnte pulmonale Parenchymlazerationen rechts mit einem ausgeprägten Hämatopneumothorax und der Verdacht auf einen Lungenhilusabriss (Abb. 2), außerdem Frakturen der ersten und zweiten Rippe beidseits. Der linke Ventrikel präsentierte sich wandverdickt.

## Falldarstellung 3

### Anamnese

Ein 21-jähriger Patient ist während seiner beruflichen Tätigkeit als Metallbauer von einer sich schließenden Dachluke am Oberkörper getroffen worden, und der Thorax wurde schließlich zwischen Luke und Fenster eingeklemmt. Nach Befreiung durch die Ersthelfer wurden unmittelbar Reanimationsmaßnahmen begonnen. Das Wiedereinsetzen des Spontankreislaufs (ROSC) blieb aus. Der Patient wurde vor Ort durch den Notarzt intubiert.

### Befund

Aufnahme in den Schockraum unter laufender kardiopulmonaler Reanimation durch die Notarztbesatzung. Die endotracheale Tubuslage war korrekt. Es zeigten sich unter Beatmung in der Auskultation vesikuläre Atemgeräusche beidseits, im EKG eine Asystolie. Die Pupillen waren weit und nicht lichtreagibel. Im E‑FAST ergab sich der Verdacht auf eine Perikardtamponade. Der Patient hatte keine weiteren offensichtlichen Extremitätenverletzungen.

### Therapie und Verlauf

Es erfolgte das Schockraummanagement entsprechend dem ATLS-Schema: Anlage von Thoraxdrainagen beidseits, mechanische Reanimationshilfe (LUCAS®, Chest Compression System, Physio-Control/Jolife AB), Erythrozyten- und Plasmatransfusionen. Im Ganzkörper-CT mit mechanischer Reanimationshilfe bestätigte sich der Verdacht auf einen Perikarderguss, weshalb eine anterolaterale Thorakotomie erfolgte. Hierbei entleerten sich etwa 2 l blutiger Perikarderguss. Ein ROSC blieb aus. Bei einer zwischenzeitlich befundeten Aortendissektion Typ A mit zudem verstrichener Mark-Rinden-Grenze und über einstündiger Reanimationszeit wurde interdisziplinär die Entscheidung getroffen, die Reanimationsmaßnahmen einzustellen.

### Bildgebung

**Figure 3:**
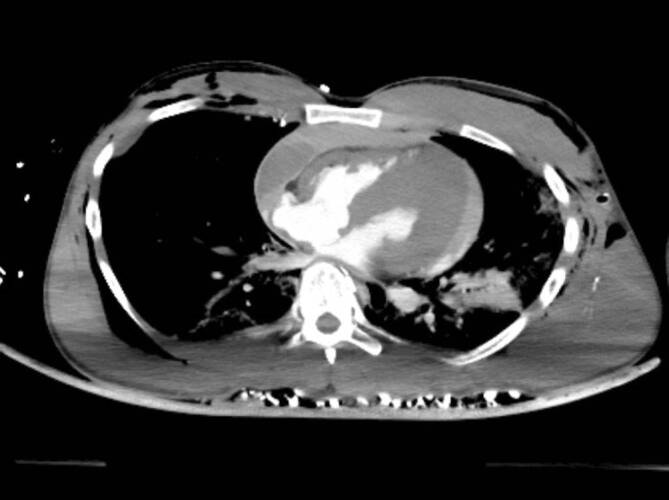

Im Ganzkörper-CT mit mechanischer Reanimationshilfe zeigten sich ein globales Hirnödem mit aufgehobener Mark-Rinden-Grenze, ein Perikarderguss und eine Aortendissektion Typ A. Außerdem zeigte sich im CT eine systolische Kontraktur des linken Ventrikels im Sinne eines Stone-heart-Phänomens (Abb. 3).

## Diskussion

Die Ursachen eines traumatisch bedingten Herz-Kreislauf-Stillstands (TCA) können Hypoxie, Spannungspneumothorax, Perikardtamponade, Hypovolämie sowie direkte strukturelle Herzverletzungen sein [4]. Ein funktioneller Herz-Kreislauf-Stillstand geht mit einem abrupten Abfall des bestehenden Blutflusses und dem Abfall des Perfusionsdrucks in den Koronarien einher [5]. Bei länger anhaltendem Kammerflimmern sinkt aufgrund der Myokardischämie die ventrikuläre Compliance [2]. Ein nichtausreichender „forward blood flow“ und eine Abnahme des Druckgradienten in der zentralen Aorta führen zu einer verminderten koronaren Perfusion [2, 6–8]. Zusätzlich kommt es durch die Freisetzung von Norepinephrin zur mikrovaskulären Vasokonstriktion und somit zur myokardialen Hypoperfusion [1]. Folge der anaeroben Stoffwechselsituation ist eine myokardiale Steifheit bzw. eine ischämische Kontraktur (auch als systolische Kontraktur in der Literatur beschrieben) entsprechend einem Stone-heart-Phänomen [9, 10].

Bei einem prolongierten Herzstillstand nimmt die Wanddicke des linken Ventrikels zu, und hierdurch reduziert sich das linksventrikuläre diastolische Volumen [2]. Währenddessen kommt es zu einer Verlagerung des Blutvolumens von der arteriellen zur venösen Seite, wodurch sich der rechte Ventrikel ausdehnt. Folgen sind eine Septumabweichung und damit eine Veränderung der kardialen Geometrie [5].

**Figure 4:**
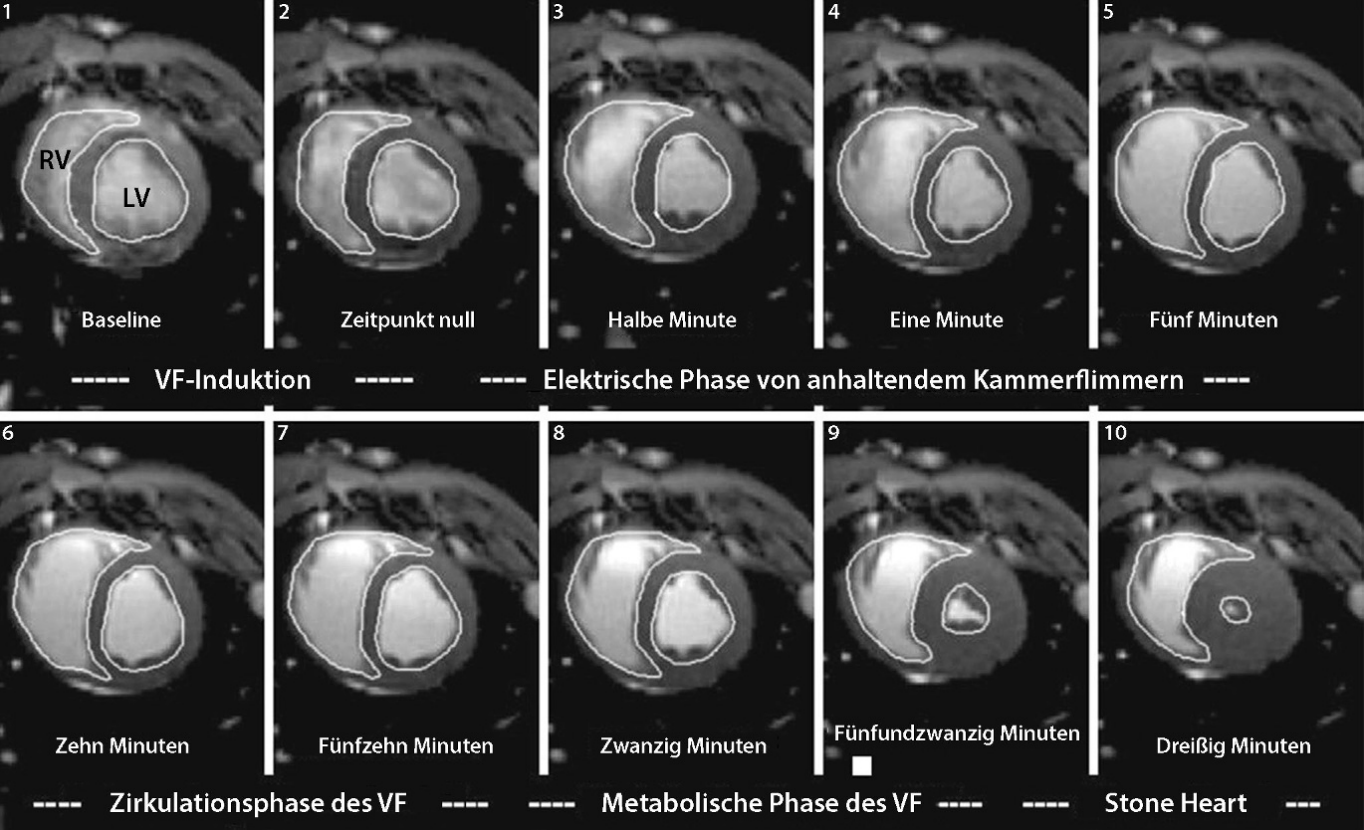

Eine schwere myokardiale Kontraktur im Sinne eines Stone-heart-Phänomens entwickelt sich während eines unbehandelten Kammerflimmers nach 25–30 min. Sorell et al. konnten dies mittels kardialer Magnetresonanztomographie (MRT) im Tiermodell zeigen (Abb. 4; [3]).

Von entscheidender Bedeutung zur Vermeidung eines Stone-heart-Phänomens ist neben der Wiederherstellung der myokardialen Oxygenierung und Perfusion das Erreichen eines „forward blood flow“. In Tierversuchsstudien konnte gezeigt werden, dass der Reanimationserfolg nach Auftreten von Kammerflimmern und Defibrillation zeitabhängig ist [2]. So wurde auch bei Patienten mit außerklinischem Herzstillstand und erfolgreicher Defibrillation mit ROSC in der elektrischen Phase (<5 min) innerhalb der ersten 3 min die höchste Überlebensrate beobachtet [11, 12]. Bei einem länger als 4 min dauernden Herzstillstand fällt die Wahrscheinlichkeit einer erfolgreichen Defibrillation drastisch ab [12]. Während der zirkulatorischen Phase (5–15 min) kommt es zu einem „shift“ des Blutes vom linken zum rechten Ventrikel. Daher sind Thoraxkompressionen in dieser Phase von großer Bedeutung, um den „forward blood flow“ zu fördern [13]. Insbesondere durch Anwendung der offenen Herzmassage kommt es zu einer deutlichen Umverteilung der Blutvolumina und damit zur Verbesserung des Blutflusses und auch des myokardialen Perfusionsdrucks [14]. Aufgrund der eingeschränkten myokardialen Compliance kann eine offene Herzdruckmassage im Vergleich zu einer „indirekten“ Thoraxkompression eine effektivere Alternative darstellen, um den systemischen, zerebralen und koronaren Perfusionsdruck zu verbessern [15]. Daraus resultiert auch eine höhere Überlebenswahrscheinlichkeit der Patienten [16–18].

In der im Schockraum durchgeführten Ganzkörpertomographie bzw. „Focused Assessment with Computed Tomography in Trauma“ (FACTT) können kardiale Geometrie, die linksventrikuläre Wanddicke und der Füllungszustand der großen Gefäße beurteilt werden. Zeigt sich hier eine deutliche linksventrikuläre Wandverdickung nach prolongiertem Herzstillstand und ohne Wiederherstellung eines Kreislaufs, kann das Einstellen der weiteren Therapie erwogen werden. Die Indikation zum Abbruch der Reanimation bei einem traumatisch bedingten Herz-Kreislauf-Stillstand ist aufgrund der nichtausreichenden Evidenz schwierig [19–21]. Wenn sich jedoch in der Bildgebung im Schockraum oder nach Notfallthorakotomie eine deutliche Wandverdickung des linken Ventrikels oder ein „steinhartes“ Herz nach prolongiertem Herzstillstand zeigt, kann man von einer länger bestehenden unzureichenden Perfusion und konsekutiver Organischämie ausgehen.

## Fazit für die Praxis

Wird ein Stone heart im Rahmen einer Computertomographie oder bei einer Notfallthorakotomie beobachtet, ist dies ein Hinweis für eine lang andauernde Myokardischämie. Man kann bei einem derartigen Befund erwägen, die Reanimationsmaßnahmen einzustellen.
